# The Radiolabeled HER3 Targeting Molecules for Tumor Imaging 

**DOI:** 10.22037/ijpr.2021.114677.14991

**Published:** 2021

**Authors:** Sajjad Molavipordanjani, Seyed Jalal Hosseinimehr

**Affiliations:** a *Department of Radiology and Nuclear Medicine, Faculty of Medicine, Cardiovascular Research Center, Mazandaran University of Medical Sciences, Sari, Iran. *; b *Department of Radiopharmacy, Faculty of Pharmacy, Mazandaran University of Medical Sciences, Sari, Iran.*

**Keywords:** HER3, PET, SPECT, Imaging, Radiolabeled, Tumor

## Abstract

The human epidermal growth factor receptor (HER) family plays pivotal roles in physiologic and pathologic conditions (such as tumor growth, proliferation, and progression in multiple epithelial malignancies). All the family members are considered tyrosine kinase, while HER3 as a member of this family shows no intrinsic tyrosine kinase. HER3 is called ‘pseudokinase’ because it undergoes heterodimerization and forms dimers such as HER2-HER3 and HER1 (EGFR)-HER3. The exact role of HER3 in cancer is still unclear; however, the overexpression of this receptor is involved in the poor prognosis of malignancies. To that end, different studies investigated the development of radiotracers for imaging of HER3. The main focus of this review is to gather all the studies on developing new radiotracers for imaging of HER3.

## Introduction

**Figure 1:**
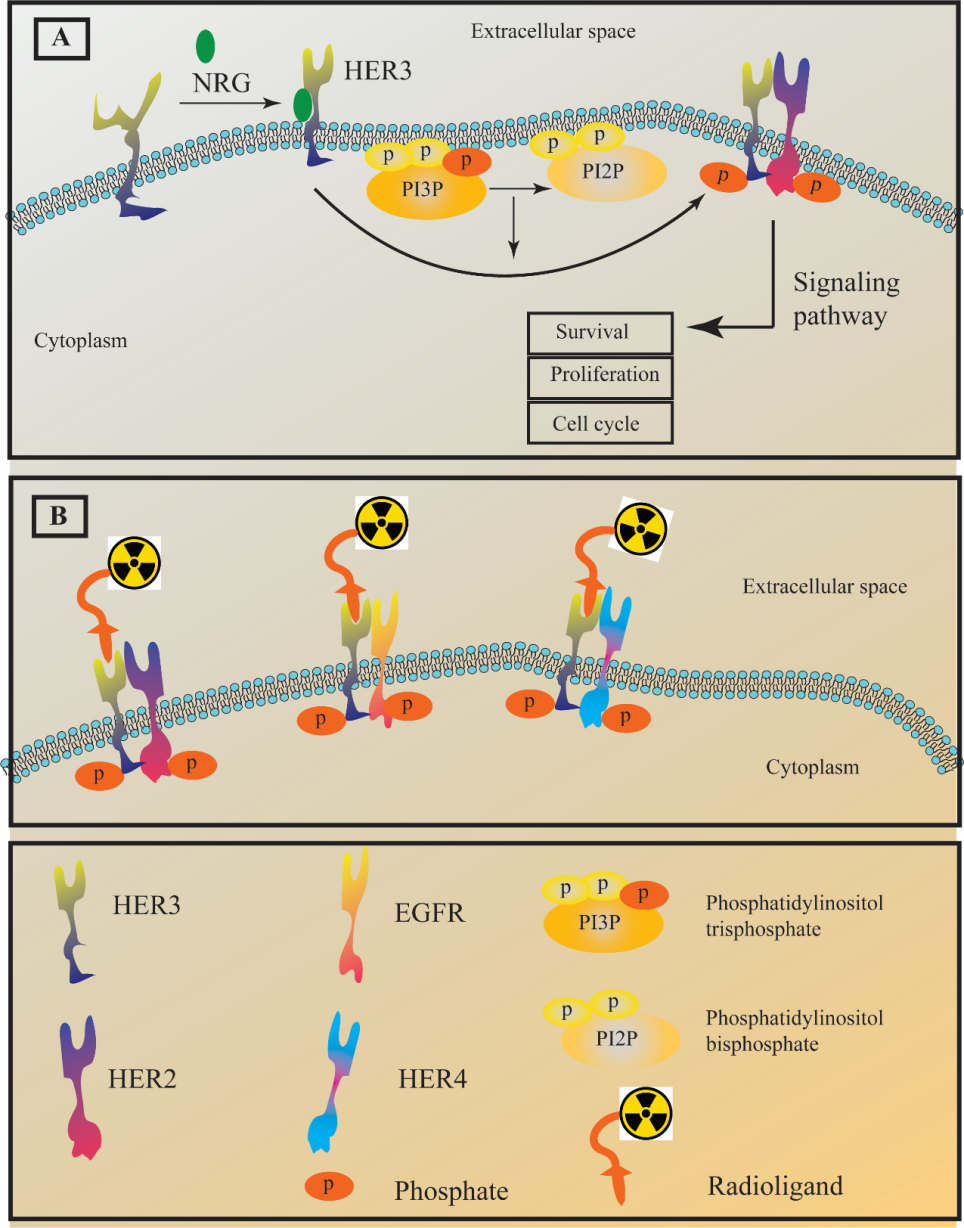
(A) The HER3 receptor adapts an active form through interaction with a ligand known as neuregulin (NRG). HER3 tyrosine kinase domain is impaired; hence, it only undergoes a weak auto-phosphorylation process. The scheme shows the hetrodimerization of HER3/HER2. However, in a similar manner it can form heterodimers with EGFR and HER4. (B) The concept behind HER3 imaging is the attachment of a radioligand to HER3 heterodimers

Epidermal growth factor receptor (EGFR) is a family of receptor tyrosine kinases which includes human epidermal growth factor receptor HER1-4 (1-4). These tyrosine kinases represent pivotal roles in different stages of epithelial malignancies such as tumor growth, proliferation, and progression (5-9). HER1 and HER2 have been targeted by approved medicine in patients diagnosed with non-small-cell lung cancer (NSCLC) and breast cancer (10, 11). The HER family members are transmembrane proteins and possess an intracellular kinase domain that can participate in cellular signaling after homodimerization through phosphorylation (12). On the contrary, HER3 shows no intrinsic tyrosine kinase properties unless it goes under hetrodimerization and forms dimers such as HER2-HER3 and HER1 (EGFR)-HER3. Having that said, HER3 is called “pseudokinase”. The lack of HER3 kinase activity directly results from mutations at the conserved residues Asp813 and Glu738. No data explains the exact role of HER3 in cancer; however its overexpression in multiple cancer types suggested HER3 as a potential target in cancer therapy (13-15). It is worth noting that different normal human tissues ranging from intestines to respiratory tracts also express this receptor. A wide range of malignancies including prostate, gastric, breast, pancreatic, lung, and ovarian cancers display overexpression of HER3 (Figure 1) (16-19). To that end, HER3-targeted cancer therapy could be an alternative to the anti-EGFR and anti-HER2 resistance therapies. Different agents such as monoclonal antibody seribantumab were investigated for cancer HER3-targeted therapy (20, 21).

Targeting HER3 also can be useful for diagnostic purposes. Different types of HER3 ligands such as short peptides, nanobodies, antibodies, antibody fragments, and affibody molecules were radiolabel and investigated for imaging purposes. The main challenge that restrains the development of HER3-imaging agents is the HER3 normal expression in several adult tissues, including the liver and gastrointestinal tracts (22, 23). Another challenge to developing an imaging radiotracer is the moderate expression of this receptor malignant tissues which is less than 5 × 10^4^ receptors/cell (24, 25). The moderate expression of this receptor in cancer tissues severely reduces the imaging contrast against the background due to the high uptake in normal tissues. The main goal of this review is to focus on the different radiotracers that were investigated for HER3-targeted imaging (Figure 1). Therefore, different aspects of HER3 radiotracers and their advantages and disadvantages will be discussed with copious details. 

**An insight into HER3 functions and structure both in normal and malignant conditions**

Like other EGFR family members, HER3 also possesses three domains, including an extracellular, a transmembrane, and an intracellular domain. The extracellular domain has four different subdomains (I-IV). The subdomains I, II, and III are involved in the ligand-binding process and it is reported that their structure is leucine-rich. On the other hand, subdomains II and IV are crucial for the receptor conformation and stability since they are cysteine-rich and can form disulfide bonds. Moreover, subunit II has a section known as the dimerization loop (26, 27). The intracellular domain of HER3 possesses different subunits, including a juxtamembrane segment, a kinase domain, and a C-terminal domain (28, 29). As mentioned earlier, subunits I and III are involved in ligand binding and binding the ligands such as neuregulins (heregulin and NRG-2), stimulats conformational change in HER3 structures and exposes the dimerization loop to other EGFR family members (30, 31). 

HER3 is expressed in the mesenchyme of the endocardial cushion, and it is closely related to the development of the heart valve. To that end, HER3 null mouse embryos displayed highly underdeveloped heart valves. HER3 involvement in neural differentiation and sympathetic nervous system development is confirmed (32-34). The heterodimer of HER3-HER2 is involved in several pathologic conditions such as resistance to different chemotherapy agents and the promotion of invasion and metastasis. Although there is no solid evidence the HER3 alone is involved in malignancies, it is worth noting that some reports claimed that the overexpression of HER2 boosts the formation of HER3-HER2 heterodimer without any need for ligand binding (neuregulins) and cause a weak but continuous signaling activity (35-37).

**Single-photon emission computed tomography (SPECT) radiotracers**

^99m^ *Tc-radiotracers*

Several γ-emitting isotopes such as ^99m^Tc (half-life 6 h) and ^111^In (half-life 67 h) for SPECT have been used to label tumor-targeting molecules for imaging purposes. Affibodies are small (7 kDa) three-helical proteins that can provide a unique tool for radionuclide imaging (38-40). To provide a proper HER3-targeted affibody-based radiotracer, an affibody with subnanomolar affinity is required. Two anti-HER3 affibody molecules (Z_08698_ and Z_08699_) have very high affinities around 50 and 21 pM, respectively (41). To that end, a histidyl-glytamyl-histidyl-glytamyl-histidyl-glytamyl-tagged Z_08699_ ((HE)_3_-Z_08699_) affibody molecule was radiolabel with ^99m^Tc(CO)_3_(H_2_O)_3_^+^. The resulted radiotracer (^99m^Tc(CO)_3_-(HE)_3_-Z_08699_) ability to target HER3 was evaluated *in-vitro* and *in-vivo.* This radiotracer displayed a high affinity toward HER3 receptors in LS174T and BT474 cell lines and xenografts (Table 1) (42). 

^111^ *In-radiotracers*

The potential of ^111^In radiolabeled Z_08698_ for HER3 imagining was also investigated. In order to provide sufficient chelator for ^111^In radiolabeling different chelators including NOTA (1,4,7-triazacyclononane-N,N′,N′′-triacetic acid), NODAGA (1-(1,3-carboxypropyl)-4,7-carboxymethyl- 1,4,7-triazacyclononane), DOTA (1,4,7,10-tetraazacyclododecane-1,4,7,10-tetraacetic acid), and DOTAGA (1,4,7,10-tetraazacyclododececane,1-(glutaric acid)−4,7,10-triacetic acid) were coupled to the C-terminus of affibody Z_08698_. This study reported that all the radiolabeled affibody including ^111^In-DOTAGA-Z_08698_, ^111^In-DOTA-Z_08698_, and ^111^In-NOTA-Z_08698_ displayed a high affinity for HER3 *in-vitro* and *in-vivo*. The tumor uptakes of all these radiotracers were the same. However, the accumulation of the negatively charged ^111^In-DOTAGA-Z_08698_ in the liver was significantly lower than the others (Table 1) (43).

A histidyl-glytamyl-histidyl-glytamyl-histidyl-glytamyl tagged Z_08699_ ((HEHEHE)-Z_08699_ or (HE)_3_- Z_08699_) affibody molecule conjugated with DOTAGA ((HE)_3_-Z_08698_-DOTAGA) was also radiolabeled with ^111^In to obtain ^111^In-(HE)_3_-Z_08698_-DOTAGA. This radiotracer possesses a high affinity toward HER3. This study also investigates the effect of non-residualizing [^125^I]-N-succinimidyl-4-iodobenzoate (^125^I-PIB) on (HE)_3_-Z_08698_-DOTAGA (^125^I-PIB-(HE)_3_-Z_08698_-DOTAGA) tumor uptake in comparison with ^111^In-(HE)_3_-Z_08698_-DOTAGA. Both of these radiotracers exhibited a high affinity toward HER3 expressing BxPC-3 and DU145 cancer cell lines. ^125^I-PIB-(HE)_3_-Z_08698_-DOTAGA showed faster clearance than ^111^In-(HE)_3_-Z_08698_-DOTAGA from most tissues, while its blood concentration was significantly higher. These radiotracers were tumor-specific, and the tumor uptake of ^125^I-PIB-(HE)_3_-Z_08698_-DOTAGA was lower. The only advantage of using non-residualizing ^125^I-PIB for affibody radiolabeling was the higher tumor-to-liver ratio (Table 1) (44). 

**Positron-emission tomography (PET) radiotracers**

^68^ *Ga-radiotracers*

The high affinity of Z_08698 _toward HER3 encouraged its radiolabeling with a wide range of radionuclides. Due to the advantages of PET such as high resolution, sensitivity, and quantification accuracy in comparison with SPECT (45,46), PET radiotracers of Z_08698_ were developed. Therefore, a chelator (NOTA) conjugated version of HEHEHE-Z_08698_ ((HE)_3_-Z_08698_) was radiolabeled with ^68^Ga (^68^Ga-(HE)_3_-Z_08698_-NOTA). This radiotracer showed a high affinity toward HER3 positive cell lines (BT474, BxPC-3) and their xenografts, while it displayed significantly lower affinity toward A431 cell line with low HER3 expression. The xenograft models of BT474, and BxPC-3 showed high tumor-to-blood and tumor-to-muscle ratios (>20 and >15, respectively) at 3 hours after injection of ^68^Ga-(HE)_3_-Z_08698_-NOTA. Therefore, the combination of PET radionuclide, high affinity toward HER3, and high tumor uptake made ^68^Ga-HEHEHE-Z_08698_-NOTA an eligible radiotracer for HER3 targeted tumor imaging (47). In another study, to reduce liver uptake of ^68^Ga-(HE)_3_-Z_08698_-NOTA, the NOTA chelator was replaced with DOTAGA. The negative charge of DOTAGA when it forms a complex with ^68^Ga could lead to lower hepatic uptake of ^68^Ga-(HE)_3_-Z_08698_-DOTAGA and better imaging properties in comparison with ^68^Ga-(HE)_3_-Z_08698_-NOTA. The hepatic uptake of ^68^Ga-(HE)_3_-Z_08698_-DOTAGA was lower compared with ^68^Ga-(HE)_3_-Z_08698_-NOTA while its blood concentration was higher; hence the quality of the imaging stayed almost the same (Table 1) (48).

In addition to ^68^Ga-affibodies, ^68^Ga-peptides are also investigated for their ability to target HER3. HER3P1 is a peptide (the sequence is CLPTKFRSC) and targets the extracellular domain of HER3. The ^68^Ga labeled HER3P1 (^68^Ga-NOTA-HER3P1) showed moderate affinity (270 ± 151 nM) toward HER3. ^68^Ga-NOTA-HER3P1 displayed affinity toward MDA-MB-453 (moderate expression of HER3) which can be blocked using excess unlabeled NOTA-HER3P1. It is worth noting that HER3P1 can distinguish HER3 from EGFR and HER2. ^68^Ga-NOTA-HER3P1 also showed high accumulation in HER3 high expressing cell line (tumor to muscle was about 1.8) 22RV1 xenograft while its accumulation in HCC-1954 (low expression of HER3) xenograft was much lower (Table 1) (49). 

^55^ *Co-* *radiotracers*

As it was mentioned earlier, (HE)_3_-Z_08698_ was radiolabeled with ^68^Ga using different chelators. However, the short half-life of ^68^Ga makes the delayed imaging almost impossible. Therefore, other PET radionuclides such as ^55^Co with a longer half-life (17.5 h) seem to be a viable option for radiolabeling (HE)_3_-Z_08698_. A study reported conjugation of different chelators such as NOTA, NODAGA, DOTA, and DOTAGA to this affibody. The resulted affibodies were radiolabeled with ^57^Co (271.8 days) as a surrogate for ^55^Co. Using different conjugated chelator results in a range of metal complexes with various charges and different RCP (radiochemical purity). All the (HE)_3_-Z_08698 _displayed high radiochemical yields (>99%) except for ^57^Co-(HE)_3_-Z_HER3:08698_-NOTA which was subject to NAP5 size-exclusion chromatography to improve purity (>99%). ^57^Co-(HE)_3_-Z_HER3:08698_-DOTA and the other conjugates specifically bind to the BxPC-3 and DU145 (HER3-expressing cell lines) which can be blocked by the addition of the cold affibody. All the conjugates display high and almost the same affinity (in subnanomolar range) toward HER3. However, ^57^Co-(HE)_3_-Z_HER3:08698_-DOTA showed the highest tumor-to- muscle ratio and the best imaging contrast at 3 and 24 h post-injection. It is noteworthy, that the application of ^57^Co as a surrogate of ^55^Co is more convenient due to its availability and longer half-life. However, the different radionuclide impurities of ^55^Co and ^57^Co may lead to different results for ^55^Co-(HE)_3_-Z_HER3:08698_-DOTA in comparison with ^57^Co-(HE)_3_-Z_HER3:08698_-DOTA (Table 1) (50). One of the drawback of ^57^Co-(HE)_3_-Z_HER3:08698_-NOTA is its high non-specific liver uptake so that co-injection of trimer affibody ((Z_HER3:08698_)_3_) with three times more molar excesses than ^57^Co-(HE)_3_-Z_HER3:08698_-NOTA concentration has been suggested to significantly reduce hepatic uptake with no impact on its tumor uptake (43).

^18^ *F-radiotracers*

Radiolabeling of a biomolecule with ^18^F usually requires a prosthetic group (51, 52). However, there are other approaches, such as using [^18^F]AlF and a chelator (NOTA) (53, 54). Sometimes a combination of these approaches can be applied. To that end, a study reported the radiolabeling of Z_HER3:8698_ affibody with ^18^F using [^18^F]AlF and a chelator (NOTA). In this study, two different methods were applied to conjugate a chelator to Z_HER3:8698_ affibody. The first approach introduced a NOTA chelator to the affibody through its cysteine residue using a maleimide functionalized NOTA (NOTA-Z_HER3:8698_) and then radiolabeled the resulted radiotracer using [^18^F]AlF to obtain [^18^F]AlF-NOTA-Z_HER3:869_. The second approach in this study was radiolabeling of tetrazine functionalized 1,4,7- triazacyclononane-1,4-diacetate (NODA) using [^18^F]AlF as the source of ^18^F to obtain a prosthetic group. The resulted molecule was then conjugated to trans-cyclooctene (TCO) functionalized Z_HER3:8698_ to synthesized [^18^F]AlF-NODA-Z_HER3:8698_. Both of the resulted radiotracers showed a high affinity toward HER3. The study indicated that these radiotracers bind to MCF-7 (high-expressing HER3 cell line), which was blocked by an excess amount of the cold affibody. Furthermore, [^18^F]AlF-NOTA-Z_HER3:869_ and [^18^F]AlF-NODA-Z_HER3:8698_ displayed very low binding to MDA-MB-231 (low expressing HER3 cell line). This study indicated that these radiotracers successfully visualized tumors in the MCF-7 xenograft model (Table 1) (55). To our personal opinion and despite the high affinity of these radiotracers toward HER3, both of these radiotracers need extensive purification after radiolabeling which is not desirable for ^18^F regarding its 110 minutes’ half-life. Besides, both of these radiotracers displayed high abdomen cavity radioactivity and high blood to tumor ratio (24.61 and 9.3 for 1 h post-injection) which jeopardizes the potential clinical application of these radiotracers. In contrast to ^89^Zr (t_1/2 _= 78.41 h), in the case of ^18^F (t_1/2 _= 109.7 min) delayed imaging cannot resolve this problem due to the much lower half-life of ^18^F in comparison with ^89^Zr. 

^89^ *Zr-radiotracers*

Radiolabeled anti-human HER3 monoclonal antibodies such as Mab#58 are also potential candidates for HER3 imaging. ^89^Zr-labeled Mab#58 was investigated as a radiotracer for imaging of HER3. Usually, radiolabeling of biomolecules requires the conjugation of a suitable bifunctional chelating agent (BFCA). Mab#58 is no exception; before radiolabeling, it was conjugated with p-isothiocyanatobenzyl-desferrioxamine B as a BFCA for ^89^Zr. The ^89^Zr-labeled Mab#58 (^89^Zr-Mab#58) displayed a high affinity for the RH7777 cell line (HER3 overexpressing cell line), and the calculated K_d_ was 2.7 nM. The biodistribution of this radiotracer showed significant tumor uptake compared to the control group. The radiotracer accumulation in the tumor tends to increase from day 1 to day 4 post-injection. This radiotracer successfully visualized the tumor in the xenograft model (Table 1) (56). However, we suspect the tumor visualization is mainly due to the radioactivity of the tumor blood content rather than tumor uptake. 

GSK2849330 is an anti HER3 monoclonal antibody. To provide sufficient chelating sites for ^89^Zr-radiolabeling p-isothiocyanatobenzyl-desferrioxamine was conjugated to this biomolecule. The final radioactive biomolecule (^89^Zr-GSK2849330) can visualize CHL-1 (HER3 expressing cell line) xenograft in mice while it does not show any accumulation in MIA-PaCa-2 (HER3 negative cell line) xenograft. This study suggests that the tumor uptake of ^89^Zr-GSK2849330 is dose-dependent, and pretreatment of CHL-1 xenograft with GSK2849330 (50 mg/kg) reduces significantly reduces tumor uptake. Confocal microscopy images also confirmed the affinity of fluorescently labeled GSK2849330 toward CHL-1 tumors. Moreover, this study expressed that GSK2849330 can inhibit tumor growth. It should be noticed that this study did not report any quantities *in-vitro* data (such as K_d_, K_i_, or IC_50_) about the affinity of ^89^Zr-GSK2849330 for HER3 (Table 1) (57). Another study reported the application of ^89^Zr-GSK2849330 in six patients diagnosed with HER3-positive tumors who were not responsive to standard treatments. The results of this study indicated that ^89^Zr-GSK2849330 displayed good tumor uptake in all six patients which their HER3 receptors were blocked with pre-treated GSK2849330. Moreover, this study reported an inhibitory dose for 50 and 90 percent (ID_50_ and ID_90_) of the patient’s population which were 2 and 18 mg/kg, respectively. However, reporting ID_50_ and ID_90 _in a population of six patients is not valid enough (58). 

A glycoengineered humanized monoclonal antibody, lumretuzumab (RG7116, RO5479599), can bind to the extracellular domain of HER3; hence it can inhibit its heterodimerization and downstream signaling pathway (59). Furthermore, a phase I clinical study in patients diagnosed via HER3-positive solid tumors indicated that they could tolerate lumretuzumab monotherapy (60). Due to this evidence, ^89^Zr-radiolabeled lumretuzumab (^89^Zr-lumretuzumab) has undergone clinical trial. ^89^Zr-lumretuzumab successfully visualized tumor lesions in patients with locally advanced or metastatic HER3-positive solid tumors. Due to the high uptake of ^89^Zr-lumretuzumab in the liver, this radiotracer failed to visualize tumor lesions in the liver while these lesions were visible on the computed tomography (CT) scan (61).

Regarding the fact that HER3-mediated resistance occurs when using Hsp90 inhibitors such as AUY922 (62, 63), Z_HER3:8698 _affibody molecule was radiolabeled with ^89^Zr not only as an imaging radiotracer but also as a tool for evaluation of HER3 expression levels in breast cancer xenograft models. To that end, first Z_HER3:8698 _was conjugated to deferoxamine-maleimide and then radiolabeled with ^89^Zr to obtain ^89^Zr-DFO-Z_HER3:8698_. The study suggests that ^89^Zr-DFO-Z_HER3:8698 _can visualize the tumor in MCF-7 xenograft. The treatment of the xenograft model with AUY922 (Hsp90 inhibitors) resulted in therapy resistance through HER3 up-regulation. To that end, the uptake of ^89^Zr-DFO-Z_HER3:8698 _in MCF-7 tumor was increased by 1.51 fold from day 0 (no AUY922) to 14 days after treatment with AUY922 (Table 1) (64). A similar study applied the ^89^Zr radiolabeled monoclonal antibody (^89^Zr-mAb3481) to evaluate the effect of lapatinib treatment on HER3 expression and the internalization of mAb3481 monoclonal antibody (65).

MEHD7945A (duligotuzumab) is a fully human IgG1 mAb that has affinity toward EGFR (K_D_ ~ 1.9 nM) and HER3 (K_D_ ~ 0.4 nM) (66,67). MEHD7945A was developed as an alternative for treating solid tumors that are resistant to EGFR-targeted treatment through HER3-mediated pathways (68,69). MEHD7945A bold characteristic in the treatment of locally advanced or metastatic epithelial cancers is its very low toxicity (70). To that end, MEHD7945A was radiolabeled with ^89^Zr (^89^Zr-MEHD7945A) for spontaneous imaging of EGFR and HER3. For *in-vitro *study of ^89^Zr-MEHD7945A three different pancreatic cell lines (Mia PACA2 (negative control), AsPC-1 and BxPC-3) with EGFR and HER3 expression were selected. The order of the HER3 expression in these cell lines is AsPC-1> BxPC-3> Mia PACA2. The uptake of ^89^Zr-MEHD7945A in these cell lines was time-dependent, and the affinity of ^89^Zr-MEHD7945A toward AsPC-1 and BxPC-3 was in the same range. This study reported that blockage of EGFR with cetuximab in AsPC-1 xenograft tumors decreased the tumor uptake of the radiotracer up to three folds while blocking the HER3 receptors with DL3.6b did not alter the radiotracer uptake. BxPC-3 xenografts blocking with cetuximab did not affect the radiotracer uptake, while the study indicated that blocking with DL3.6b lead to increasing the radiotracer uptake. In the end, the author of this study suggested that the expression of HER3 in comparison with EGFR is five-fold lower (using flow cytometry data) in both AsPC-1 and BxPC-3. Therefore, the combination of marginal changes in DL3.6b blocked HER3, and a higher level of EGFR expression may put the changes below the sensitivity threshold of ^89^Zr-MEHD7945A (Table 1) (71). In our personal view, it seems that ^89^Zr-MEHD7945A is an EGFR imaging probe rather than HER3.

MSB0010853 is a nanobody that is capable of binding to two different epitopes of HER3. Therefore, it can be a robust platform for the development of a HER3 targeted radiotracer. To that end, a study reported ^89^Zr radiolabeled MSB0010853 (^89^Zr-MSB0010853) for PET imaging of HER3 receptors. ^89^Zr-MSB0010853 successfully recognized the HER3 receptors of the H441 xenografted model in a dose-dependent manner which was blocked using an excess amount of cold MSB0010853. This study indicated that after 24 hours (post-injection) the accumulation of the radiotracer in the tumor reached its maximum and the tumor site in H441 and FaDu (HER3 positive cell line) xenografted was visualized while the tumor site in Calu-1 (negative HER3 cell line) is not observable (Table 1) (72). It should be noticed that the MSB0010853 nanobody contains a binding site for albumin and can cross-reactive with albumin and HER3. Moreover, the tumor and blood radioactivity uptakes at 24 h post-injection are very close together. Therefore, to our personal opinion, it is not clear how much of tumor uptake is related to the interaction of HER3 and ^89^Zr-MSB0010853 and how much is related to tumor blood content. 

^11^ *C- radiotracers*

AZD8931 ((73)) is a known small molecule with a tyrosine kinase inhibitory effect. This molecule possesses equipotent affinity toward EGFR, HER2, and HER3 (74, 75). Therefore, it was radiolabeled with ^11^C, ([^11^C]AZD8931), to image HER3 receptors. The high affinity of [^11^C]AZD8931 toward HER3 and high specific activity made it a potential radiotracer for PET imaging of HER3 (Table 1) (73). 

**Table 1:** A list of HER3 radiotracers and their characteristics

**No.**	**Radiotracer**	**RCP (%)**	**Type of molecule**	**Imaging modality**	**Affinity**	**Specific activity**	**Cell line**	**Tumor to muscle ratio**	**Tumor to blood ratio**	**Tumor to liver ratio**	**Ref.**
2	^99m^Tc(CO)_3_-(HE)_3_-Z_08699_	>99	Affibody	SPECT	K_D_ = 27 pM	1.6 MBq/μg	LS174T and BT474	11-12 (4 h p.i.)^a^	6-7 (4 h p.i.)	<0.5 (4 h p.i.)	(42)
3	^111^In-DOTAGA-Z_08698_	>99	Affibody	SPECT	K_D_ = 8 ± 6 pM	-	BxPC-3 and DU145	21 (24 h p.i.)	6 (4 h p.i.)	0.61 (4 h p.i.)	(43)
4	^111^In-(HE)_3_-Z_08698_-DOTAGA	>99	Affibody	SPECT	K_D_ = 19 ± 1 pM	1.2 MBq/μg	BxPC-3 and DU145	1.63 (4 h p.i.)	43 (4 h p.i.)	0.7 (4 h p.i.)	(44)
5	^68^Ga-(HE)_3_-Z_08698_-NOTA	>98	Affibody	PET	-	16–19 GBq/μmol	LS174T, BxPC-3, BT474	>15 (3 h p.i.)	2 (1 h p.i.)	0.95 (1 h p.i.)	(47)
6	^68^Ga-NOTA-HER3P1	>95	Peptide	PET	K_D_ = 270 ± 151 nM	296 ± 25.9 MBq/mg	22RV1	1.8 (1 h p.i.)	2.78 (1 h p.i.)	0.71 (1 h p.i.)	(49)
7	^57^Co-(HE)_3_-Z_HER3:08698_-DOTA	99.7	Affibody	SPECT	K_D_ = 0.4 ± 0.8 nM	-	BxPC-3 and DU145	28±4 (24 h p.i.)	18 (24 h p.i.)	1.6 (24 h p.i.)	(50)
8	[^18^F]AlF-NOTA-Z_HER3:869_	>98	Affibody	PET	K_D_ = 0.44 ± 0.04 nM	0.8-1.5 MBq	MCF-7	34.96 (1 h p.i.)	24.61 (1 h p.i.)	0.77 (1 h p.i.)	(55)
9	[^18^F]AlF-NODA-Z_HER3:8698_	>95	Affibody	PET	K_D_ = 1.01 ± 0.28 nM	0.7-2.3 MBq/μg	MCF-7	18.01(1 h p.i.)	9.27 (1 h p.i.)	0.86 (1 h p.i.)	(55)
10	^89^Zr-Mab#58	>90	Monoclonal antibody	PET	K_D_ = 2.7 nM	40–110 kBq/μg	RH7777	>10 (4 days p.i.)	1.1 (4 days p.i.)	1.3 (4 days p.i.)	(56)
11	^89^Zr-DFO-Z_HER3:8698_	>95	Affibody	PET	K_D_ = 0.55 ± 0.05 nM	2.5 - 2.7 MBq/μg	MCF-7	20.4 (3 h p.i.)	4.75 (3 h p.i.)	1.2 (3 h p.i.)	(64)
12	^89^Zr-MEHD7945A	>95	Monoclonal antibody	PET	IC_50_ = 0.37-0.48 nM	25.5 ± 3.7 MBq/nmol	BxPC-3 and AsPC-1	>20 (96 h p.i.)	>6 (96 h p.i.)	>5 (96 h p.i.)	(71)
13	^89^Zr-MSB0010853	>95	Nanobody	PET	-	-	H441 and FaDu	>10 (24 h p.i.)	8 (24 h p.i.)	<1 (24 h p.i.)	(72)
14	[^11^C]AZD8931	>99	Small molecule	PET	IC_50_ = 4 nM	370–1110 GBq/µmol	-	-	-	-	(73)

^a^p.i; post-injection.

## Conclusion

The exact role of the HER3 receptor in malignancies is still unclear. However, this receptor is certainly linked to therapy resistance and poor prognosis of some cancers; hence, trying to develop a clinically useful radiotracer for imaging of this radiotracer seems reasonable. Due to a few difference between HER3 and other members of the human epidermal growth factor receptor family, such as HER2 developing radiotracer for HER3 imaging is much more challenging because: firstly; even in the case of HER3 overexpression in cancer, the number of HER3 does not exceed 50000 receptors/cell and, secondly; normal human tissues, such as the respiratory, urinary, gastrointestinal, and reproductive tract and even skin physiologically express HER3 receptor. To that end, to develop a radiotracer for HER3, the affinity of the tracer should be very high toward HER3 (preferably in the picomolar range), such as different radiotracer of Z_HER3:08698 _affibody. Furthermore, such radiotracers are more effective when they are radiolabel with longer half-life radionuclide. To that end, the ^89^Zr (t_1/2 _= 78.4 h) radiotracers are much more desirable and practical in comparison with ^18^F (t_1/2 _= 109.7 min) radiotracer, due to the fact that delayed imaging with ^89^Zr radiotracer is possible. 

## Conflict of interest

The authors declare no conflict of interest. 

## References

[1] Sigismund S, Avanzato D, Lanzetti L (2018). Emerging functions of the EGFR in cancer. Mol. Oncol..

[2] Mitchell RA, Luwor RB, Burgess AW (2018). Epidermal growth factor receptor: Structure-function informing the design of anticancer therapeutics. Exp. Cell Res..

[3] Wang Z (2017). ErbB Receptors and Cancer. Methods Mol. Biol..

[4] Pool M, de Boer HR, Hooge MNL, van Vugt M, de Vries EGE (2017). Harnessing integrative omics to facilitate molecular imaging of the human epidermal growth factor receptor family for precision medicine. Theranostics.

[5] An Z, Aksoy O, Zheng T, Fan QW, Weiss WA (2018). Epidermal growth factor receptor and EGFRvIII in glioblastoma: signaling pathways and targeted therapies. Oncogene.

[6] Liu X, Wang P, Zhang C, Ma Z (2017). Epidermal growth factor receptor (EGFR): A rising star in the era of precision medicine of lung cancer. Oncotarget.

[7] Wee P, Wang Z (2017). Epidermal Growth Factor Receptor Cell Proliferation Signaling Pathways. Cancers (Basel).

[8] Byeon HK, Ku M, Yang J (2019). Beyond EGFR inhibition: multilateral combat strategies to stop the progression of head and neck cancer. Exp. Mol. Med..

[9] Matsumoto K, Umitsu M, De Silva DM, Roy A, Bottaro DP (2017). Hepatocyte growth factor/MET in cancer progression and biomarker discovery. Cancer Sci..

[10] Rebuzzi SE, Alfieri R, La Monica S, Minari R, Petronini PG, Tiseo M (2020). Combination of EGFR-TKIs and chemotherapy in advanced EGFR mutated NSCLC: Review of the literature and future perspectives. Crit. Rev. Oncol. Hematol..

[11] Rotow J, Bivona TG (2017). Understanding and targeting resistance mechanisms in NSCLC. Nat. Rev. Cancer.

[12] Nami B, Maadi H, Wang Z (2019). The effects of pertuzumab and its combination with trastuzumab on HER2 homodimerization and phosphorylation. Cancers (Basel).

[13] Lim SM, Xie T, Westover KD, Ficarro SB, Tae HS, Gurbani D, Sim T, Marto JA, Jänne PA, Crews CM, Gray NS (2015). Development of small molecules targeting the pseudokinase Her3. Bioorg. Med. Chem. Lett..

[14] Xie T, Lim SM, Westover KD, Dodge ME, Ercan D, Ficarro SB, Udayakumar D, Gurbani D, Tae HS, Riddle SM, Sim T, Marto JA, Jänne PA, Crews CM, Gray NS (2014). Pharmacological targeting of the pseudokinase Her3. Nat. Chem. Biol..

[15] Claus J, Patel G, Ng T, Parker PJ (2014). A role for the pseudokinase HER3 in the acquired resistance against EGFR- and HER2-directed targeted therapy. Biochem. Soc. Trans..

[16] Li QH, Wang YZ, Tu J, Liu CW, Yuan YJ, Lin R, He WL, Cai SR, He YL, Ye JN (Oxf). Anti-EGFR therapy in metastatic colorectal cancer: mechanisms and potential regimens of drug resistance. Gastroenterol. Rep..

[17] Dieci MV, Miglietta F, Griguolo G, Guarneri V (2020). Biomarkers for HER2-positive metastatic breast cancer: Beyond hormone receptors. Cancer Treat. Rev..

[18] Maennling AE, Tur MK, Niebert M, Klockenbring T, Zeppernick F, Gattenlöhner S, Meinhold-Heerlein I, Hussain AF (2019). Molecular targeting therapy against EGFR family in breast cancer: progress and future potentials. Cancers (Basel).

[19] Arienti C, Pignatta S, Tesei A (2019). Epidermal growth factor receptor family and its role in gastric cancer. Front. Oncol..

[20] Liu X, Liu S, Lyu H, Riker AI, Zhang Y, Liu B (2019). Development of effective therapeutics targeting HER3 for cancer treatment. Biol. Proced. Online.

[21] Karachaliou N, Lazzari C, Verlicchi A, Sosa AE, Rosell R (2017). HER3 as a therapeutic target in cancer. Biodrugs.

[22] Prigent SA, Lemoine NR, Hughes CM, Plowman GD, Selden C, Gullick WJ (1992). Expression of the c-erbB-3 protein in normal human adult and fetal tissues. Oncogene.

[23] Srinivasan R, Poulsom R, Hurst HC, Gullick WJ (1998). Expression of the c-erbB-4/HER4 protein and mRNA in normal human fetal and adult tissues and in a survey of nine solid tumour types. J. Pathol..

[24] Bae SY, La Choi Y, Kim S, Kim M, Kim J, Jung SP, Choi MY, Lee SK, Kil WH, Lee JE, Nam SJ (2013). HER3 status by immunohistochemistry is correlated with poor prognosis in hormone receptor-negative breast cancer patients. Breast. Cancer Res. Treat..

[25] Ho-Pun-Cheung A, Bazin H, Boissière-Michot F, Mollevi C, Simony-Lafontaine J, Landas E, Bleuse JP, Chardès T, Prost JF, Pèlegrin A, Jacot W, Mathis G, Lopez-Crapez E (2020). Quantification of HER1, HER2 and HER3 by time-resolved Förster resonance energy transfer in FFPE triple-negative breast cancer samples. Br. J. Cancer.

[26] Cho HS, Leahy DJ (2002). Structure of the extracellular region of HER3 reveals an interdomain tether. Science.

[27] Kani K, Park E, Landgraf R (2005). The extracellular domains of ErbB3 retain high ligand binding affinity at endosome pH and in the locked conformation. Biochemistry.

[28] Brand TM, Iida M, Luthar N, Wleklinski MJ, Starr MM, Wheeler DL (2013). Mapping C-terminal transactivation domains of the nuclear HER family receptor tyrosine kinase HER3. PLoS One.

[29] Choi BK, Cai X, Yuan B, Huang Z, Fan X, Deng H, Zhang N, An Z (2012). HER3 intracellular domains play a crucial role in HER3/HER2 dimerization and activation of downstream signaling pathways. Protein Cell.

[30] Büttner R, Berndt A, Valkova C, Richter P, Korn A, Kosan C, Liebmann C (2017). Myofibroblasts have an impact on expression, dimerization and signaling of different ErbB receptors in OSCC cells. J. Recept. Signal Transduct. Res..

[31] Collier TS, Diraviyam K, Monsey J, Shen W, Sept D, Bose R (2013). Carboxyl group footprinting mass spectrometry and molecular dynamics identify key interactions in the HER2-HER3 receptor tyrosine kinase interface. J. Biol. Chem..

[32] Miyamoto Y, Torii T, Tanoue A, Kawahara K, Arai M, Tsumura H, Ogata T, Nagao M, Terada N, Yamamoto M, Takashima S, Yamauchi J (2017). Neuregulin-1 type III knockout mice exhibit delayed migration of Schwann cell precursors. Biochem. Biophys. Res. Commun..

[33] Espinosa-Medina I, Jevans B, Boismoreau F, Chettouh Z, Enomoto H, Müller T, Birchmeier C, Burns AJ, Brunet JF (2017). Dual origin of enteric neurons in vagal Schwann cell precursors and the sympathetic neural crest. Proc. Natl. Acad. Sci. U. S. A.

[34] Steiner H, Blum M, Kitai ST, Fedi P (1999). Differential expression of ErbB3 and ErbB4 neuregulin receptors in dopamine neurons and forebrain areas of the adult rat. Exp. Neurol..

[35] Taieb J, Jung A, Sartore-Bianchi A, Peeters M, Seligmann J, Zaanan A, Burdon P, Montagut C, Laurent-Puig P (2019). The evolving biomarker landscape for treatment selection in metastatic colorectal cancer. Drugs.

[36] Black LE, Longo JF, Carroll SL (2019). Mechanisms of receptor tyrosine-protein kinase ErbB-3 (ERBB3) Action in human neoplasia. Am. J. Pathol..

[37] Citri A, Skaria KB, Yarden Y (2003). The deaf and the dumb: the biology of ErbB-2 and ErbB-3. Exp. Cell Res..

[38] Ståhl S, Gräslund T, Eriksson Karlström A, Frejd FY, Nygren P, Löfblom J (2017). Affibody Molecules in Biotechnological and Medical Applications. Trends Biotechnol..

[39] Frejd FY, Kim KT (2017). Affibody molecules as engineered protein drugs. Exp. Mol. Med..

[40] Tolmachev V, Orlova A (2020). Affibody molecules as targeting vectors for PET Imaging. Cancers (Basel).

[41] Malm M, Kronqvist N, Lindberg H, Gudmundsdotter L, Bass T, Frejd FY, Höidén-Guthenberg I, Varasteh Z, Orlova A, Tolmachev V, Ståhl S, Löfblom J (2013). Inhibiting HER3-mediated tumor cell growth with affibody molecules engineered to low picomolar affinity by position-directed error-prone PCR-like diversification. PLoS One.

[42] Orlova A, Malm M, Rosestedt M, Varasteh Z, Andersson K, Selvaraju RK, Altai M, Honarvar H, Strand J, Ståhl S, Tolmachev V, Löfblom J (2014). Imaging of HER3-expressing xenografts in mice using a (99m)Tc(CO) 3-HEHEHE-Z HER3:08699 affibody molecule. Eur. J. Nucl. Med. Mol. Imaging.

[43] Rosestedt M, Andersson KG, Rinne SS, Leitao CD, Mitran B, Vorobyeva A, Ståhl S, Löfblom J, Tolmachev V, Orlova A (2019). Improved contrast of affibody-mediated imaging of HER3 expression in mouse xenograft model through co-injection of a trivalent affibody for in-vivo blocking of hepatic uptake. Sci. Rep..

[44] Rinne SS, Xu T, Dahlsson Leitao C, Ståhl S, Löfblom J, Orlova A, Tolmachev V, Vorobyeva A (2020). Influence of residualizing properties of the radiolabel on radionuclide molecular imaging of HER3 using affibody molecules. Int. J. Mol. Sci..

[45] Thomas M, Patel KK, Peri-Okonny P, Sperry BW, McGhie AI, Badarin FA, Saeed IM, Kennedy KF, Chan P, Spertus JA, Thompson RC, Bateman TM (2020). Stress myocardial perfusion imaging in patients presenting with syncope: Comparison of PET vs. SPECT. J. Nucl. Cardiol..

[46] Spencer SS, Theodore WH, Berkovic SF (1995). Clinical applications: MRI, SPECT, and PET. Magn. Reson. Imaging.

[47] Rosestedt M, Andersson KG, Mitran B, Tolmachev V, Löfblom J, Orlova A, Ståhl S (2015). Affibody-mediated PET imaging of HER3 expression in malignant tumours. Sci. Rep..

[48] Rinne SS, Dahlsson Leitao C, Gentry J, Mitran B, Abouzayed A, Tolmachev V, Ståhl S, Löfblom J, Orlova A (2019). Increase in negative charge of (68)Ga/chelator complex reduces unspecific hepatic uptake but does not improve imaging properties of HER3-targeting affibody molecules. Sci. Rep..

[49] Larimer BM, Phelan N, Wehrenberg-Klee E, Mahmood U (2018). Phage Display Selection, In-vitro characterization, and correlative PET imaging of a novel HER3 peptide. Mol. Imaging Biol..

[50] Rinne SS, Dahlsson Leitao C, Saleh-Nihad Z, Mitran B, Tolmachev V, Ståhl S, Löfblom J, Orlova A (2020). Benefit of later-time-point PET imaging of HER3 expression using optimized radiocobalt-labeled affibody molecules. Int. J. Mol. Sci..

[51] Perrin DM (2018). Organotrifluoroborates as prosthetic groups for single-step F18-labeling of complex molecules. Curr. Opin. Chem. Biol..

[52] Schirrmacher R, Wängler B, Bailey J, Bernard-Gauthier V, Schirrmacher E, Wängler C (2017). Small Prosthetic Groups in (18)F-Radiochemistry: Useful auxiliaries for the design of (18)F-PET tracers. Semin. Nucl. Med..

[53] Fersing C, Bouhlel A, Cantelli C, Garrigue P, Lisowski V, Guillet B (2019). A comprehensive review of non-covalent radiofluorination approaches using aluminum [(18)F]fluoride: will [(18)F]AlF replace (68)Ga for metal chelate labeling?. Molecules.

[54] Kumar K, Ghosh A (2018). (18)F-AlF labeled peptide and protein conjugates as positron emission tomography imaging pharmaceuticals. Bioconjug. Chem..

[55] Da Pieve C, Allott L, Martins CD, Vardon A, Ciobota DM, Kramer-Marek G, Smith G (2016). Efficient [(18)F]AlF radiolabeling of ZHER3:8698 affibody molecule for imaging of HER3 positive tumors. Bioconjug. Chem..

[56] Yuan Q, Furukawa T, Tashiro T, Okita K, Jin ZH, Aung W, Sugyo A, Nagatsu K, Endo H, Tsuji AB (2015). Immuno-PET imaging of HER3 in a model in which HER3 signaling plays a critical role. PLoS One.

[57] Alsaid H, Skedzielewski T, Rambo MV, Hunsinger K, Hoang B, Fieles W, Long ER, Tunstead J, Vugts DJ, Cleveland M (2017). Non invasive imaging assessment of the biodistribution of GSK2849330, an ADCC and CDC optimized anti HER3 mAb, and its role in tumor macrophage recruitment in human tumor-bearing mice. PLoS One.

[58] Menke-van der Houven van Oordt CW, McGeoch A, Bergstrom M, McSherry I, Smith DA, Cleveland M, Al-Azzam W, Chen L, Verheul H, Hoekstra OS, Vugts DJ, Freedman I, Huisman M, Matheny C, van Dongen G, Zhang S (2019). Immuno-PET imaging to assess target engagement: experience from 89Zr-Anti-HER3 mAb (GSK2849330) in patients with solid tumors. J. Nucl. Med..

[59] Mirschberger C, Schiller CB, Schräml M, Dimoudis N, Friess T, Gerdes CA, Reiff U, Lifke V, Hoelzlwimmer G, Kolm I, Hopfner KP, Niederfellner G, Bossenmaier B (2013). RG7116, a therapeutic antibody that binds the inactive HER3 receptor and is optimized for immune effector activation. Cancer Res..

[60] Meulendijks D, Jacob W, Martinez-Garcia M, Taus A, Lolkema MP, Voest EE, Langenberg MHG, Fleitas Kanonnikoff T, Cervantes A, De Jonge MJ, Sleijfer S, Soerensen MM, Thomas M, Ceppi M, Meneses-Lorente G, James I, Adessi C, Michielin F, Abiraj K, Bossenmaier B, Schellens JHM, Weisser M, Lassen UN (2016). First-in-human phase i study of lumretuzumab, a glycoengineered humanized anti-HER3 monoclonal antibody, in patients with metastatic or advanced HER3-positive solid tumors. Clin. Cancer Res..

[61] Bensch F, Lamberts LE, Smeenk MM, Jorritsma-Smit A, Lub-de Hooge MN, Terwisscha van Scheltinga AGT, de Jong JR, Gietema JA, Schröder CP, Thomas M, Jacob W, Abiraj K, Adessi C, Meneses-Lorente G, James I, Weisser M, Brouwers AH, de Vries EGE (2017). 89Zr-lumretuzumab PET imaging before and during HER3 antibody lumretuzumab treatment in patients with solid tumors. Clin. Cancer Res..

[62] Augello G, Emma MR, Cusimano A, Azzolina A, Mongiovì S, Puleio R, Cassata G, Gulino A, Belmonte B, Gramignoli R, Strom SC, McCubrey JA, Montalto G, Cervello M (2019). Targeting HSP90 with the small molecule inhibitor AUY922 (luminespib) as a treatment strategy against hepatocellular carcinoma. Int. J. Cancer.

[63] Ishikawa C, Senba M, Mori N (2016). Efficiency of AUY922 in mice with adult T-cell leukemia/lymphoma. Oncol. Lett..

[64] Martins CD, Da Pieve C, Burley TA, Smith R, Ciobota DM, Allott L, Harrington KJ, Oyen WJG, Smith G, Kramer-Marek G (2018). HER3-mediated resistance to Hsp90 inhibition detected in breast cancer xenografts by affibody-based PET imaging. Clin. Cancer Res..

[65] Pool M, Kol A, de Jong S, de Vries EGE, Lub-de Hooge (2017). MN and Terwisscha van Scheltinga AGT (89)Zr-mAb3481 PET for HER3 tumor status assessment during lapatinib treatment. MAbs.

[66] De Pauw I, Wouters A, Van den Bossche J, Deschoolmeester V, Baysal H, Pauwels P, Peeters M, Vermorken JB, Lardon F (2017). Dual targeting of epidermal growth factor receptor and HER3 by MEHD7945A as monotherapy or in combination with cisplatin partially overcomes cetuximab resistance in head and neck squamous cell carcinoma cell Lines. Cancer Biother. Radiopharm..

[67] Jimeno A, Machiels JP, Wirth L, Specenier P, Seiwert TY, Mardjuadi F, Wang X, Kapp AV, Royer-Joo S, Penuel E, McCall B, Pirzkall A, Clement PM (2016). Phase Ib study of duligotuzumab (MEHD7945A) plus cisplatin/5-fluorouracil or carboplatin/paclitaxel for first-line treatment of recurrent/metastatic squamous cell carcinoma of the head and neck. Cancer.

[68] Schaefer G, Haber L, Crocker Lisa M, Shia S, Shao L, Dowbenko D, Totpal K, Wong A, Lee Chingwei V, Stawicki S, Clark R, Fields C, Lewis Phillips Gail D, Prell Rodney A, Danilenko Dimitry M, Franke Y, Stephan J-P, Hwang J, Wu Y, Bostrom J, Sliwkowski Mark X, Fuh G, Eigenbrot C (2011). A two-in-one antibody against HER3 and EGFR Has superior inhibitory activity compared with monospecific antibodies. Cancer Cell.

[69] Bourillon L, Demontoy S, Lenglet A, Zampieri A, Fraisse J, Jarlier M, Boissière-Michot F, Perrochia H, Rathat G, Garambois V, Bonnefoy N, Michaud H-A, Chardès T, Tosi D, Pèlegrin A, Azria D, Larbouret C, Bourgier C (2020). Higher anti-tumor efficacy of the Dual HER3-EGFR Antibody MEHD7945a combined with ionizing irradiation in cervical cancer cells. Int. J. Radiat. Oncol. Biol. Phys..

[70] Juric D, Dienstmann R, Cervantes A, Hidalgo M, Messersmith W, Blumenschein GR, Tabernero J, Roda D, Calles A, Jimeno A, Wang X, Bohórquez SS, Leddy C, Littman C, Kapp AV, Shames DS, Penuel E, Amler LC, Pirzkall A, Baselga J (2015). Safety and pharmacokinetics/pharmacodynamics of the first-in-class dual action HER3/EGFR antibody MEHD7945A in locally advanced or metastatic epithelial tumors. Clin.l Cancer Res..

[71] McKnight BN, Kuda-Wedagedara ANW, Sevak KK, Abdel-Atti D, Wiesend WN, Ku A, Selvakumar D, Carlin SD, Lewis JS, Viola-Villegas NT (2018). Imaging EGFR and HER3 through 89Zr-labeled MEHD7945A (Duligotuzumab). Sci. Rep..

[72] Warnders FJ, Terwisscha van Scheltinga AGT, Knuehl C, van Roy M, de Vries EFJ, Kosterink JGW, de Vries EGE, Lub-de Hooge MN (2017). Human epidermal growth factor receptor 3-specific tumor uptake and biodistribution of (89)Zr-MSB0010853 visualized by real-time and noninvasive PET imaging. J. Nucl. Med..

[73] Wang M, Gao M, Zheng Q-H (2014). The first radiosynthesis of [11C]AZD8931 as a new potential PET agent for imaging of EGFR, HER2 and HER3 signaling. Bioorg. Med. Chem. Lett..

[74] Adams R, Brown E, Brown L, Butler R, Falk S, Fisher D, Kaplan R, Quirke P, Richman S, Samuel L, Seligmann J, Seymour M, Shiu KK, Wasan H, Wilson R, Maughan T (2018). Inhibition of EGFR, HER2, and HER3 signalling in patients with colorectal cancer wild-type for BRAF, PIK3CA, KRAS, and NRAS (FOCUS4-D): a phase 2-3 randomised trial. Lancet Gastroenterol. Hepatol..

[75] Barlaam B, Anderton J, Ballard P, Bradbury RH, Hennequin LF, Hickinson DM, Kettle JG, Kirk G, Klinowska T, Lambert-van der Brempt C, Trigwell C, Vincent J, Ogilvie D (2013). Discovery of AZD8931, an equipotent, reversible inhibitor of signaling by EGFR, HER2, and HER3 receptors. ACS Med. Chem. Lett..

